# Genetic Polymorphisms of *Interleukin-16* and Risk of Knee Osteoarthritis

**DOI:** 10.1371/journal.pone.0123442

**Published:** 2015-05-08

**Authors:** Shi-Xing Luo, Shan Li, Xue-Hui Zhang, Jun-Jing Zhang, Guang-Hua Long, Gui-Fu Dong, Wei Su, Yan Deng, Yanqiong Liu, Jin-Min Zhao, Xue Qin

**Affiliations:** 1 Department of Orthopedic Trauma and Hand Surgery, The First Affiliated Hospital of Guangxi Medical University, Nanning, Guangxi, China; 2 Department of Trauma Orthopedics, Ninth Affiliated Hospital of Guangxi Medical University, Beihai, Guangxi, China; 3 Department of Clinical Laboratory, First Affiliated Hospital of Guangxi Medical University, Nanning, Guangxi, China; 4 Department of Nuclear medicine, Ninth Affiliated Hospital of Guangxi Medical University, Beihai, Guangxi, China; 5 Graduate school of Guangxi Medical University, Nanning, Guangxi, China; Garvan Institute of Medical Research, AUSTRALIA

## Abstract

**Background:**

Interleukin-16 (IL-16), a pleiotropic cytokine, plays a fundamental role in inflammatory diseases. This study investigates the association between *IL-16* polymorphisms and the risk of knee osteoarthritis (OA) in a Chinese population.

**Methods:**

The *IL-16 rs11556218*, *rs4072111*, and *rs4778889* polymorphisms were determined in 150 knee OA cases and 147 healthy controls through polymerase chain reaction-restriction fragment length polymorphism.

**Results:**

The results suggested that the variants in *IL-16* gene *rs11556218* site were associated with a decreased knee OA risk after adjusting for age, sex, BMI, and smoking and drinking status (TG vs. TT: OR, 0.69; 95% CI, 0.53–0.89; *P* = 0.006; GG vs. TT: OR, 0.64; 95% CI, 0.45–0.90; *P* = 0.042; dominant model: OR, 0.68; 95% CI, 0.29–0.87; *P* = 0.002; G vs. T allele: OR, 0.77; 95% CI, 0.66–0.90; *P* = 0.003). Similarly, subjects bearing the *rs4072111* variant genotypes and alleles also had a lower susceptibility to knee OA compared with those bearing the wild-type (CT vs. CC: OR, 0.66; 95% CI, 0.53–0.83; *P* = 0.002; TT vs. CC: OR, 0.57; 95% CI, 0.40–0.82; *P* = 0.027; dominant model: OR, 0.65; 95%, CI 0.52–0.80; *P* <0.001; T vs. C allele: OR, 0.69; 95% CI, 0.58–0.81; *P* <0.001). Further, the C allele and the combined genotype (CC+CT) of *rs4778889* were associated with a slightly decreased risk of knee OA. In addition, we found two high-risk haplotypes: TTT (OR, 3.70) and GCC (OR, 6.22). Finally, serum IL-16 levels of knee OA patients were significantly higher than those of controls (*P* = 0.001).

**Conclusions:**

Despite the small sample size, this is the first study suggesting *IL-16* gene polymorphisms to be associated with the risk of knee OA.

## Introduction

Osteoarthritis (OA) of the knee, which affects about 10% of adults over 55 years old, is a common but complex disease characterized by the degradation of articular cartilage, often resulting in joint disability [1]. Although many risk factors have been associated with OA, including age, previous injury, obesity, diet, hormone therapy, and smoking habits [2–4], the pathogenesis of OA remains largely unknown and needs to be further elucidated.

Inflammatory processes and cytokines play essential roles in the pathogenesis of synovitis and cartilage destruction associated with OA [5, 6]. Variations in cytokine levels among individuals are a plausible explanation for differences in disease susceptibility and severity, and are principally attributable to single nucleotide polymorphisms (SNPs) in cytokine-encoding genes [7]. This relationship is particularly true for cytokine gene polymorphisms and OA; previous studies have investigated the relationship between a series of cytokines, such as *interleukin* (*IL*)*-1* [8], *IL-4* [9], *IL-6* [7], *IL-17* [10], *IL-18* [11], and tumor necrosis factor-alpha (*TNF-α*) [12] gene polymorphisms, and the risk of developing OA. However, these genes can explain only a small part of the genetic component of this complex disease.

IL-16, as a pro-inflammatory cytokine whose functions include chemoattraction and modulation of T cell activation [13], is an important mediator in inflammatory and autoimmune diseases, as well as in tumor growth and progression [14, 15]. The *IL-16* gene is located on chromosome 15q26.3 [16] and is initially translated into a precursor protein consisting of 631 amino acids, which is cleaved by caspase-3 to form the active C-terminal domain containing 121 amino acids [17, 18]. IL-16 is a CD4-specific ligand required for the initiation of CD4 bioactivity. Through binding to the CD4 molecule, IL-16 can selectively activate CD4+ T cells, monocytes, macrophages, eosinophils, and dendritic cells [19, 20]. In addition, IL-16 can increase the production of inflammatory cytokines, such as TNF-α, IL-1β, IL-6, and IL-15, leading to inflammatory response [21, 22]. Thus, it is biologically reasonable to hypothesize a potential relationship between *IL-16* gene polymorphisms and knee OA risk.

Several *IL-16* gene SNPs have been thoroughly investigated. A common SNP in *IL-16* gene is *rs4778889* T/C, located 295 bp upstream from the transcription start site and associated with altered levels of gene expression [23]. Another two SNPs, *rs11556218* T/G and *rs4072111* C/T, are located in an exon region, and their single-nucleotide changes would result in an amino acid substitution; the first results in an asparagine (*Asn*) to lysine (*Lys*) substitution in exon 6 of the *IL-16* gene, and the second represents a serine (*Ser*) to proline (*Pro*) substitution. Several studies have recently revealed that *IL-16* gene polymorphisms are associated with several human diseases, including gastric cancer [24], colorectal cancer [25], renal cell carcinoma [26], Graves’ disease [27], coronary heart disease [28], and ischemic stroke [29]. We have previously identified a significant association between the *rs11556218* T/G polymorphism of the *IL-16* gene and susceptibility to hepatocellular [30] and nasopharyngeal carcinoma [31] in a Chinese population.However, to date, there have been no reports on the relationship of *IL-16* gene polymorphisms and knee OA. The aim of the present study was to analyze the association of *IL-16* polymorphisms with knee OA susceptibility and the influence of SNPs on IL-16 serum levels in patients with knee OA versus healthy controls in a Chinese population.

## Materials and Methods

### Study subjects

This case-control study was approved by the ethics committee of the First Affiliated Hospital of Guangxi Medical University, China. All of the participants provided written informed consent.

A total of 150 patients diagnosed with primary knee OA and 147 healthy controls were consecutively selected from the First Affiliated Hospital of Guangxi Medical University and the Ninth Affiliated Hospital of Guangxi Medical University in Guangxi, China, between February 2011 and February 2013. Knee OA diagnosis was evaluated according to the American College of Rheumatology clinical criteria [32]. The following exclusion criteria were considered: rheumatoid arthritis, ankylosing spondylitis, septic arthritis, and other arthritis or any other systemic inflammatory or autoimmune disorders. Further, patients with a previous traumatic knee injury or any history of trauma were excluded from the study. An alcohol drinker was defined as someone who consumed alcoholic beverages at least once per week for more than 6 months. Subjects were considered smokers if they smoked up to 1 year before the date of diagnosis for cases, or up to the date of interview for controls.

The controls without clinical evidence of OA and any disease mentioned as exclusion criteria were randomly selected from a pool of healthy volunteers who visited the general health check-up centers at the same hospitals during the same time period for routine scheduled physical exams.

### DNA extraction

Peripheral blood samples (2 mL) were collected from all of the subjects in ethylenediaminetetraacetic acid-coated vials and stored at—20°C until DNA extraction. Genomic DNA was extracted from white blood cell fractions using the phenol-chloroform extraction method. DNA concentration was determined spectrophotometrically.

### Genotyping of the *IL-16* genomic variants

The *rs11556218*, *rs4072111*, and *rs4778889* polymorphism genotypes were determined by the polymerase chain reaction-restriction fragment length polymorphism (PCR-RFLP) method. The primer sequences, reaction conditions, restriction enzymes used, and length of digestion products are listed in Table 1. To confirm the genotyping results, a total of 30 (10%) PCR-amplified DNA samples were randomly selected and genotyped by DNA sequencing with an ABI Prism 3100 (Applied Biosystems, Shanghai Sangon Biological Engineering Technology & Services Co., Ltd., China). The results were 100% concordant.

**Table 1:** Primer sequences and reaction conditions for genotyping *IL-16* polymorphisms.

Polymorphisms	Primer sequence 5’–>3’	Annealing temperature (℃)	Restriction enzyme	Digestion product length (bp)
rs11556218T/G	F: GCTCAGGTTCACAGAGTGTTTCCATA; R: TGTGACAATCACAGCTTGCCTG	61.0	*Nde* I	TT: 147+24; TG: 171+147+24; GG: 171
rs4072111C/T	F: CACTGTGATCCCGGTCCAGTC; R: TTCAGGTACAAACCCAGCCAGC	67.0	*BsmA* I	CC:164; CT: 164+140+24; TT:140+24
rs4778889T/C	F: CTCCACACTCAAAGCCTTTTGTTCCTATGA; R: CCATGTCAAAACGGTAGCCTCAAGC	63.0	*Ahd* I	TT: 280; TC: 280+246+34; CC: 246+34

### Serum IL-16 levels

Serum samples were available for all patients and healthy controls. Following blood sample collection, the serum was allowed to clot for 30 min at 4°C before centrifugation at 3,000 rpm for 10 min at 4°C. Total serum was isolated and stored at—20°C until further use. Serum IL-16 concentrations were detected using a sandwich ELISA with the same batch of reagents according to the manufacturer’s instructions. The minimum level of detection for IL-16 was 5 pg/mL. The intra-assay coefficients of variation were 10%.

### Sample size consideration

We estimated the sample size using Quanto software (version 1. 2.4). We based on probability of α = 0.05 and β = 0.1 and assuming that the prevalence of the risk allele (*rs11556218* G) in the control group was 40% [30], and estimated odds ratio (OR) was 0.5 Approximately 1 to 1 case–control ratio was chosen. According to the above parameters, estimated 134 sample size had enough power to assess the effect of *IL-16* genetic polymorphisms on the risk of knee OA.

### Statistical analysis

Student’s *t*-test (for continuous variables) or χ^2^ test (for categorical variables) were used to evaluate differences in the distributions of selected demographic variables, and frequencies of genotypes of *IL-16* polymorphisms between cases and controls. Agreement with the Hardy-Weinberg equilibrium for each SNP was tested using a goodness-of-fit χ^2^ test. Genotype, allele, and haplotype distributions of *IL-16* were compared among different groups using the χ^2^ test and Fisher’s exact test when appropriate. The Haploview software [33] was used to calculate the degree of pairwise linkage disequilibrium (LD) for each pair of SNPs as well as for haplotype analysis. We developed binary logistic regression models to estimate odds ratios (ORs) with corresponding 95% confidence intervals (CIs) to test the association of the various genotypes of interest and the risk of knee OA. All ORs were adjusted for age, gender, BMI, smoking and drinking state. Statistical significance was assumed at two-sided *P* values at <0.05 level. All of the statistical analyses were performed in the Statistical Package for Social Sciences (SPSS, version 13.0).

## Results

### Characteristics of the study population

Table 2 summarizes the characteristics of the 150 knee OA patients and 147 control subjects included in this study. The mean ages (SD) of the control group and knee OA group were 58.3 ± 9.6 and 59.5 ± 8.9 years, respectively. There were no significant differences for sex, mean age, BMI, smoking and drinking status between cases and control groups, suggesting that subjects matching based on these variables was adequate.

**Table 2:** Demographic characteristics of the study population.

Variables	Healthy control (n = 147) n(%)	Knee osteoarthritis patients (n = 150) n(%)	*P* value
**Age(mean±SD)**	58.3±9.6	59.5±8.9	0.13
**Gender**			
Male	51	41	0.07
Femle	96	109	
**Body mass index (kg/m** ^**2**^ **)**	23.7 ± 2.5	24.2 ± 3.3	0.19
**Smoking**			
No	118 (80.3%)	129(86.0%)	0.187
Yes	29 (19.7%)	21(14.0%)	
**Drinking**			
No	121(82.3%)	119 (79.3%)	0.514
Yes	26 (17.7%)	31 (20.7%)	
**IL-16 concentration ($\bar{\text{x}}$±S, pg/mL)**	36.70±6.72	44.32±8.78	0.001

### Genotype and allele distribution of *IL-16* polymorphisms

The distribution of each allele and genotype is shown in Table 3. All three SNPs were within the Hardy-Weinberg equilibrium. For the *rs11556218* polymorphism, there was a significant difference in the genotype and allele frequencies among knee OA patients and control subjects. The frequencies of the TT, TG, and GG genotypes of *rs11556218* were 36.7%, 51.1%, and 12.2% in healthy controls, and 55.3%, 37.3%, and 7.4% in patients with knee OA, respectively. Binary logistic regression analyses adjusting for age, gender, BMI, smoking and drinking status showed that the TG and GG genotypes of *rs11556218* were both associated with a statistically significant decreased risk of knee OA compared with the TT genotype (OR, 0.69; 95% CI, 0.53–0.89; *p* = 0.006 for TG genotype; OR, 0.64; 95% CI, 0.45–0.90; *p* = 0.042 for GG genotype). Under the dominant model, the combined genotypes GG + TG appeared to have lower susceptibility to OA (OR = 0.68, 95% CI 0.29–0.87, *p* = 0.002). The data also revealed that subjects with the G allele appeared to have a lower susceptibility to knee OA compared with those bearing the T allele (OR, 0.58; 95% CI, 0.41–0.82; *p* = 0.002).

**Table 3:** Distributions of *IL-16* SNPs genotypes in each group and logistic regression analyses of associations between these polymorphisms and knee OA risk.

Genotypes	Overall				Women				Men			
	Controls n = 147(%)	OA cases n = 150(%)	OR (95% CI)^a^	*p*	Controls n = 96	OA cases n = 109	OR (95% CI)^b^	*p*	Controls n = 51	OA cases n = 41	OR (95% CI)^b^	*p*
*rs11556218*												
TT	54 (36.7)	83(55.3)	1.00^ref^		34	61	1.00^ref^		20	22	1.00^ref^	
TG	75(51.1)	56(37.3)	0.69(0.53–0.89)	0.006	51	41	0.65(0.47–0.89)	0.011	24	15	0.77(0.52–1.16)	0.301
GG	18(12.2)	11(7.4)	0.64(0.45–0.90)	0.042	11	7	0.59(0.37–0.92)	0.047	7	4	0.75(0.43–1.29)	0.544
Dominant model												
TT	54	83	1.00^ref^		34	61	1.00^ref^		20	22	1.00^ref^	
GG+TG	93	67	0.68(0.29–0.87)	0.002	62	48	0.64(0.46–0.87)	0.005	31	19	0.79(0.52–1.13)	0.241
Recessive model												
TG+TT	129	139	1.00^ref^		85	102	1.00^ref^		44	37	1.00^ref^	
GG	18	11	0.78(0.57–1.06)	0.219	11	7	0.74(0.50–1.11)	0.306	7	4	0.85(0.52–1.39)	0.795
T allele	183 (62.2)	222 (74.0)	1.00^ref^		119	163	1.00ref		64	59	1.00^ref^	
G allele	111 (37.8)	78(26.0)	0.77(0.66–0.90)	0.003	73	55	0.74(0.60–0.91)	0.007	38	23	0.84(0.65–1.08)	0.246
*rs4072111*												
CC	89 (60.5)	120(80.0)	1.00ref		61	88	1.00^ref^		28	32	1.00^ref^	
CT	49(33.3)	27(18.0)	0.66(0.53–0.83)	0.002	29	19	0.68(0.50–0.91)	0.029	20	8	0.65(0.46–0.93)	0.048
TT	9 (6.1)	3(2.0)	0.57(0.40–0.82)	0.027	6	2	0.82(0.30–2.22)	0.718	3	1	0.62(0.33–1.17)	0.561
Dominant model												
CC	89	120	1.00^ref^		61	88	1.00^ref^		29	32	1.00^ref^	
TT+CT	58	30	0.65(0.52–0.80)	<0.001	35	21	0.66(0.50–0.87)	0.009	23	9	0.66(0.47–0.93)	0.043
Recessive model												
CT+CC	138	147	1.00^ref^		90	107	1.00^ref^		48	40	1.00^ref^	
TT	9	3	0.31(0.08–1.18)	0.071	35	21	0.73(0.57–0.94)	0.039	3	1	0.73(0.40–1.32)	0.771
C allele	227(75.7)	267(89.0)	1.00^ref^		151	195	1.00^ref^		76	72	1.00^ref^	
T allele	67(23.3)	33(11.0)	0.69(0.58–0.81)	<0.001	41	23	0.68(0.55–0.85)	0.004	26	10	0.71(0.55–0.92)	0.038
*rs4778889*												
TT	82 (55.8)	101 (67.3)	1.00^ref^		50	71	1.00^ref^		32	30	1.00^ref^	
TC	56 (38.1)	43 (28.7)	0.79(0.63–1.00)	0.079	37	35	0.80(0.59–1.10)	0.226	19	8	0.73(0.52–1.03)	0.158
CC	9 (6.1)	6 (4.0)	0.75(0.48–1.16)	0.387	9	3	0.55(0.37–0.82)	0.041	0	3	6.46(0.37–11.62)	0.248
Dominant model												
TT	82	101	1.00^ref^		50	71	1.00^ref^		32	30	1.00^ref^	
CC+TC	65	49	0.79(0.63–0.98)	0.045	46	38	0.76(0.57–1.10)	0.079	19	11	0.62(0.57–1.17)	0.403
Recessive model												
TT+TC	138	144	1.00^ref^		87	106	1.00^ref^		51	38	1.00^ref^	
CC	9	6	0.82(0.53–1.25)	0.569	9	3	0.60(0.42–0.86)	0.086	0	3	2.43(0.34–4.54)	0.170
T allele	220(74.8)	245(81.7)	1.00^ref^		137	177	1.00^ref^		83	68	1.00^ref^	
C allele	74(25.2)	55(18.3)	0.83(0.69–0.98)	0.047	55	41	0.76(0.62–0.94)	0.026	19	14	0.96(0.69–1.32)	0.936

*OA*, osteoarthritis, *OR* odds ratio, *CI* confidence interval, *ref* reference

**Bold** indicated the difference was significant.

^a^Adjusted for age, sex, smoking and drinking status by logistic regression model.

^b^ Adjusted for age, smoking and drinking status by logistic regression model.

Regarding the *rs4072111* polymorphism, the frequencies of the CC, CT, and TT genotypes were 60.5%, 33.3%, and 6.1% for control subjects and 80%, 18%, and 2% in knee OA patients, respectively. The CT and TT genotypes were associated with a significantly decreased risk of knee OA compared with patients with the CC genotype (OR, 0.66; 95% CI, 0.53–0.83; *p* = 0.002 and OR, 0.57; 95% CI, 0.40–0.82; *p* = 0.027, respectively). The combined CC+TC genotypes were also associated with a significantly decreased risk of knee OA (OR, 79; 95% CI, 063–0.98; *P* = 0.045). Using the C allele as a reference, a significant correlation was detected between the presence of the T allele and a lower risk of developing knee OA (OR, 0.69; 95% CI, 0.58–0.81; *p* <0.001).

For genotype and allele frequencies of the *IL-16 rs4778889* T/C polymorphisms, we found that subjects with the C allele and combined CC+TC genotypes (dominant model) appeared to have a slightly lower risk of knee OA compared with those bearing the T allele (OR, 0.68; 95% CI, 0.45–0.99; *p* = 0.044, and OR, 0.79; 95% CI, 0.63–0.98; *P* = 0.044, respectively).

### Stratified analysis

When analyses of genotype and allele frequencies were stratified by gender, significant differences in the distributions of *IL-16* polymorphisms among patients with knee OA and control groups were observed (Table 3). Women who carried the *IL-16* (*rs11556218* T/G) G allele had a significantly decreased risk of knee OA compared with those carrying the T allele (OR, 0.74; 95% CI, 0.60–0.91; *p* = 0.007). Similarly, women who carried the *IL-16* (*rs4072111* C/T) T allele showed a lower susceptibility to knee OA compared with those carrying the C allele (OR, 0.68; 95% CI, 0.55–0.85; *p* = 0.004). Men who carried the *IL-16* (*rs4072111* C/T) T allele showed a decreased risk of knee OA compared with those carrying the C allele (OR, 0.71; 95% CI, 0.55–0.92; *p* = 0.038), but no significant differences were found for the *rs11556218* T/G polymorphism. Regarding the *rs4778889* SNP, we found a significant difference of the genotype and allele frequencies between knee OA patients and controls in women, but not in men.

### Haplotype analyses of *IL-16* gene polymorphisms and knee OA risk

LD analyses were performed in knee OA patients and healthy controls using the Haploview ver.4.2 software. No statistically significant evidence of LD was observed among these three SNPs between knee OA patients and healthy controls (for *rs11556218* and *rs4072111*, *D’* = 0.22, r^2^ = 0.006; for *rs4778889* and *rs11556218*, *D’* = 0.59, r^2^ = 0.195; for *rs4778889* and *rs4072111*, *D’* = 0.64, r^2^ = 0.030). Further, haplotype analysis to evaluate the haplotype frequencies of polymorphisms located within the same chromosome regions was performed in order to derive haplotypes specifically correlated with knee OA. A total of seven haplotypes were derived from the observed genotypes. The haplotype distributions in knee OA patients and healthy controls are shown in Table 4. Two high-risk haplotypes were found: TTT (OR, 3.70; 95% CI, 1.57–8.72; *p* = 0.003) and GCC (OR, 6.22; 95% CI, 2.37–16.33; *p* = 0.0004). The remaining haplotypes were not associated with risk of knee OA.

**Table 4:** Haplotype analysis between the case and control groups.

Haplotypes	SNPs			Haplotype analyses		
	rs11556218	rs4072111	rs4778889	Total frequency	OR (95% CI)	*P* value
TCT	T	C	T	0.58	1.00*	—
GCT	G	C	T	0.14	1.58 (0.93–2.68)	0.093
TCC	T	C	C	0.12	1.09 (0.56–2.13)	0.800
TTT	T	T	T	0.10	3.70 (1.57–8.72)	0.003
GCC	G	C	C	0.04	6.22 (2.37–16.33)	0.0004
GTT	G	T	T	0.04	2.73 (0.54–13.87)	0.230
GTC	G	T	C	0.01	3.72 (0.46–29.96)	0.220

### Serum IL-16 levels and polymorphisms

The median serum concentration of IL-16 detected was 36.70 ± 6.72 pg/mL in healthy controls and 44.32 ± 8.78 pg/mL in knee OA patients (Table 2). The serum levels of IL-16 detected in knee OA patients were significantly higher than those in healthy control subjects (*p* = 0.001). However, when studying the relationship between the *IL-16* polymorphisms present and IL-16 serum levels among patients with knee OA and healthy controls, no significant differences were observed.

## Discussion

In the present study, we selected a common *IL-16* SNP, namely *rs4778889*, located 295 bp upstream from the transcription start site and associated with altered levels of gene expression [23], as well as two other SNPs (*rs11556218* and *rs4072111*), to evaluate their association in patients with knee OA and healthy controls. The latter two SNPs (*rs11556218* and *rs4072111*) are located in an exon region, and their single-nucleotide changes would result in an amino acid substitution. To the best of our knowledge, this is the first study to investigate whether *IL-16* gene polymorphisms are associated with the risk of knee OA and whether these correlate with serum levels of IL-16. The present results revealed that the *IL-16 rs11556218* polymorphism, representing an *Asn* to *Lys* substitution in exon 6 of the *IL-16* gene, has a significant effect on the risk of knee OA; individuals carrying the *rs11556218* G allele had a significantly decreased risk of developing knee OA compared with those carrying the T allele (OR, 0.77; 95% CI, 0.66–0.90). In addition, the non-synonymous SNP, *rs4072111* C/T, representing a *Ser* to *Pro* substitution, was associated with a significantly decreased risk of developing knee OA. Regarding the *IL-16 rs4778889* T/C polymorphism, we found that subjects with the C allele appeared to have a slightly lower risk of knee OA compared with those bearing the T allele (OR, 0.83; 95% CI, 0.69–0.98). We further evaluated the effect of *IL-16* polymorphisms on knee OA risk stratified by sex and found that the association appeared stronger in female patient subgroups.

Despite the positive relationship between *rs11556218* and *rs4072111* polymorphisms and the risk of knee OA observed in this study, IL-16 serum levels did not show any significant differences other than being cumulatively higher in patients with knee OA relative to healthy controls. In addition, we found two haplotypes (TTT and GCC) to be significantly associated with susceptibility to knee OA. Thus, the above data indicates that there is no association between *IL-16* polymorphisms and IL-16 serum levels. However, the results also suggest that *IL-16* gene polymorphisms may be significantly associated with the risk of knee OA. Although the sample size is not large enough, this is the first case-control study evaluating the association between *IL-16* polymorphisms and the risk of knee OA.

Previous attempts have been made to identify genetic factors involved in knee OA using genome-wide association studies (GWAS). Recently, several OA susceptibility loci have been identified in GWAS with significance levels [34–40]. Among them, the *DVWA* gene (SNPs *rs11718863* and *rs7639618*) and a region containing HLA class II/III genes (SNPs *rs7775228* and *rs10947262*). However, this genome-wide significant association was shown in Asians but not in Europeans [34–36]. On the other hand, genome-wide significant loci were identified to have an association with knee OA in Europeans. These included the *rs3815148* SNP in *COG5* on chromosome 7q22 [37, 38], *rs11842874* in *MCF2L* on chromosome 13 [40], and *rs6976* in *GNL3* on chromosome 3 [39]. To date, the *IL-16* gene has not yet been identified through GWAS. In fact, recent GWAS in knee OA mainly focused on chromosome 3, chromosome 7, and chromosome 13 genes [34, 37–39]. The *IL-16* gene is located on chromosome 15q26.3. IL-16 is a pleiotropic cytokine whose functions include chemoattraction and modulation of T cell activation [13] and is an important mediator in inflammatory and autoimmune diseases as well as in tumor growth and progression [14, 15]. In addition, *IL-16* can activate the secretion of tumor-associated inflammatory cytokines, such as TNF-α, IL-1β, IL-6, and IL-15, all of which are major factors involved in tumorigenesis [21, 22]. In recent years, *IL-16* gene polymorphisms have been associated with several human diseases, including gastric cancer [24], colorectal cancer [25], renal cell carcinoma [26], Graves’ disease [27], coronary heart disease [28, 41, 42], and ischemic stroke [29]. Our previous study has shown that the *IL-16 rs11556218* T/G polymorphism was significantly associated with susceptibility to both hepatocellular [30] and nasopharyngeal carcinoma [31].

Pathologically, various inflammatory components are involved in OA. In OA, the increased synthetic and anti-inflammatory activity of chondrocytes loses out to the increased degradative activity [43, 44]. The increased synthetic activity is confined to the deeper cartilage layers, which allows the imbalance towards degradation to persist in the upper layer, near the synovial boundary. Ultimately, chondrocyte malfunction and apoptosis limit the response potential and hasten the progression of OA [43, 44]. Therefore, we postulated that *IL-16* polymorphisms may modulate the susceptibility to OA.

In the current study, we found that patients carrying the G (*rs11556218* T/G), T (*rs4072111* C/T), and C (*rs4778889* T/C) alleles were associated with a significantly decreased risk for knee OA compared to individuals carrying the wild-type alleles. This finding is consistent with our hypothesis, suggesting that *IL-16 rs11556218*, *rs4072111*, and *rs4778889* polymorphisms may play an important role in the pathogenesis of knee OA. Further, we found that the association between *IL-16 rs11556218* T/G and *rs4778889* T/C polymorphisms and knee OA risk appeared stronger in female patients. Nevertheless, this evidence is suggestive but not conclusive, and was unexpected and difficult to explain. It is possible that this result is due to the larger number of female subjects (n = 205) compared to male subjects (n = 92), resulting in a limited statistical power and robustness. On the other hand, it might be attributed to the lower exposure to risk factors, such as tobacco smoking and heavy drinking, of female patients compared to males. Finally, this association might also be the result of estrogen-related effects; estrogen can interact with IL-16 and reduce the possibility of developing knee OA [45–47]. Nevertheless, since the sample size of the current study was relatively small, these findings need to be confirmed by further larger sample size studies which also investigate the underlying mechanisms of this association.

A haplotype is a set of SNPs on a single chromatid which are likely to be inherited together in a block pattern more frequently than expected by chance owing to the presence of linkage disequilibrium [48]. In the current study, we found that two haplotypes (TTT and GCC) of the *IL-16* gene were significantly associated with the susceptibility to knee OA (OR, 3.70; 95% CI, 1.57–8.72 for the TTT haplotype and OR, 6.22; 95% CI, 2.37–16.33 for the GCC haplotype). We also evaluated the influence of *IL-16* polymorphisms on IL-16 serum levels in patients with knee OA versus healthy controls, and found that the median serum level of IL-16 in patients was significantly higher than in controls. Our data suggest that a higher serum IL-16 level might serve as a risk factor for knee OA. However, the serum levels of IL-16 detected in groups of patients with different genotypes did not show any significant differences, probably due to the relatively small sample size; further confirmation would be provided by additional patient data.

Several potential limitations of this study must be acknowledged. First, our patient sample size was relatively small and therefore the study’s statistical power may have been limited. Thus, additional studies with larger samples are desirable. Second, the study population was limited to the Guangxi population and therefore the findings may not be generalized to other populations. Continued study of the role of *IL-16* polymorphisms in patient susceptibility to knee OA from other ethnic populations would also be of great value. Finally, the current research studied only three SNPs in the *IL-16* gene. It would be interesting to identify more SNPs and study their association with knee OA.

In conclusion, the present study showed that functional polymorphisms of *IL-16* are associated with the risk of knee OA. We found that the variant alleles *rs11556218* T/G, *rs4072111*C/T, and *rs4778889* T/C were associated with a decreased risk of knee OA compared with wild-type alleles. These findings suggest that the *IL-16 rs11556218* T/G, *rs4072111* C/T, and *rs4778889* T/C polymorphisms might be markers for genetic susceptibility to knee OA. Furthermore, serum IL-16 levels were significantly associated with increased risk of knee OA. These findings, after validation by larger studies, might help identify at-risk populations for primary knee OA prevention.
